# Mechanical and Durability Characteristics of Particulate-Filled Recycled Thermoplastic Composites (RTCs): A Comprehensive Review

**DOI:** 10.3390/polym17233161

**Published:** 2025-11-27

**Authors:** Md Sabbrojjaman, Allan Manalo, Wahid Ferdous, Omar Alajarmeh

**Affiliations:** 1 Centre for Future Materials, University of Southern Queensland, Toowoomba, QLD 4350, Australia; md.sabbrojjaman@unisq.edu.au; 2 School of Engineering, Centre for Future Materials, University of Southern Queensland, Toowoomba, QLD 4350, Australia; wahid.ferdous@unisq.edu.au; 3 Department of Civil and Environmental Engineering, United Arab Emirates University, Al Ain P.O. Box 15551, United Arab Emirates; omar.alajarmeh@unisq.edu.au

**Keywords:** recycled thermoplastic composites, particulate fillers, mechanical, durability

## Abstract

Globally, over 350 million tonnes of thermoplastic waste are generated annually, with more than 60% either landfilled or mismanaged. This attracts innovative pathways to increase their recyclability, among which particulate-filled recycled thermoplastic composites (RTCs) are emerging as a potential waste reuse strategy for diverse civil and industrial applications. This review systematically analyses the current understanding of the physical, mechanical, and durability performance of RTCs, focusing on how various particulate filler types, content, and interfacial compatibility influence key properties. Reported studies show that incorporating particulate organic or inorganic fillers such as waste glass, sand, wood flour, etc., can increase density by 10–45%, tensile and flexural moduli by 30–120%, and thermal stability by up to 40%, though strength and ductility often decrease by 15–50% due to poor filler–matrix adhesion. This review further evaluates durability enhancements under prolonged exposure to water, thermal, and UV radiation, where filler addition reduces water absorption and UV degradation by 20–60%. Despite these advancements, challenges remain in optimising interfacial bonding, long-term performance modelling, and scalability for civil infrastructure. This review also outlines research directions to advance high-performance, sustainable RTCs through a structured review approach using defined keywords on recycled thermoplastics, fillers, and durability.

## 1. Introduction

**Figure 1:**
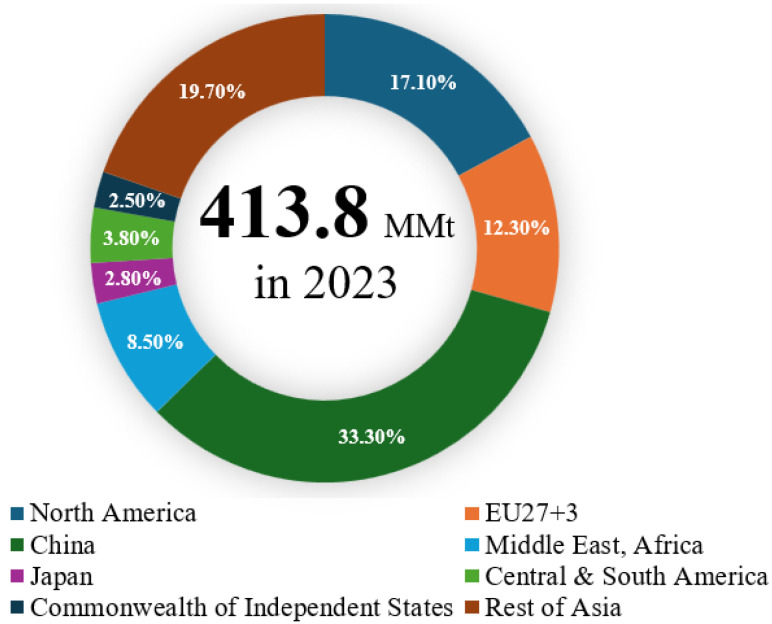
Total global plastics production over time, illustrating the rapid increase in plastic manufacturing worldwide and the resulting growth in plastic waste generation [3].

**Figure 2:**
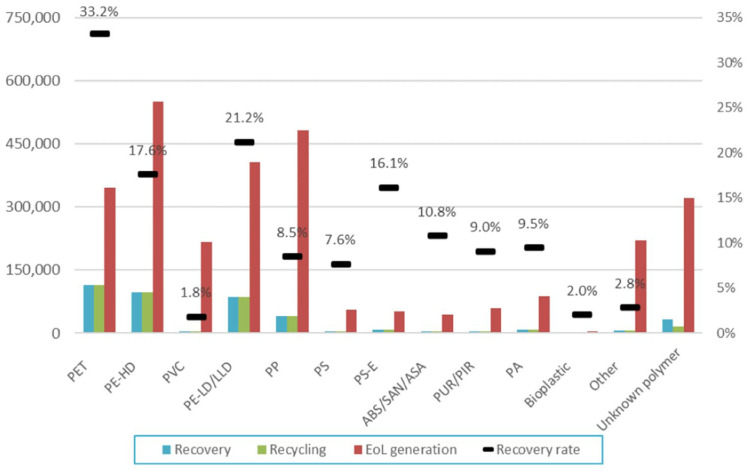
Plastics recovery, recycling, and EoL generation in Australia, 2021–22 (tonnes) [6].

Thermoplastics are the second-highest source of municipal waste globally, amounting to approximately 413.8 million tonnes (Mt) in 2023 [1], following food and fruit waste [2]. The details of total global plastics production are shown in Figure 1 [3]. Additionally, the volume of plastic wastes doubled over the past two decades, with more than 60% ending up in landfills or unmanaged environments, resulting in economic losses estimated between USD 80 and 120 billion annually due to the value of plastics lost after a single use [4]. Projections indicate that by 2050, approximately 12,000 Mt of plastic waste will be disposed of in natural bodies or landfills [5]. The Australian Government Department’s “National Waste and Resource Recovery Report 2024” revealed that Australia generated an estimated 3.0 Mt of plastics during the financial year 2022–23, despite a national plastics recycling rate of 13.9% in 2021–22. The report of plastics recovery, recycling, and end-of-life (EoL) generation by polymer type in Australia in 2021–22 is presented in Figure 2 [6].

However, repeated melt cycles during thermoplastic recycling can lead to chain scission, oxidative degradation, and loss of stabilisers, significantly reducing their tensile strength, impact resistance, and thermal stability compared to virgin polymers [7,8]. Environmental ageing and long-term heat exposure during the service life of thermoplastics further deteriorate their performance [9,10].

Several studies have focused on enhancing the performance of recycled thermoplastics by incorporating particulate fillers [11,12,13]. Fillers such as organic, inorganic, mineral particulates, agricultural residues, and nano-reinforcements have been used to partially restore stiffness, improve dimensional stability, and enhance thermal resistance [14]. While these strategies have shown success in specific applications, limitations persist—including filler agglomeration, poor dispersion, incompatibility with degraded polymer chains, and the sample becoming brittle or processing difficulties at high loadings [15,16]. These challenges highlight the need to explore new filler systems—particularly those derived from sustainable, waste-based, or functionalised nano-materials—that can achieve balanced property enhancement without compromising processability or durability.

**Figure 3:**
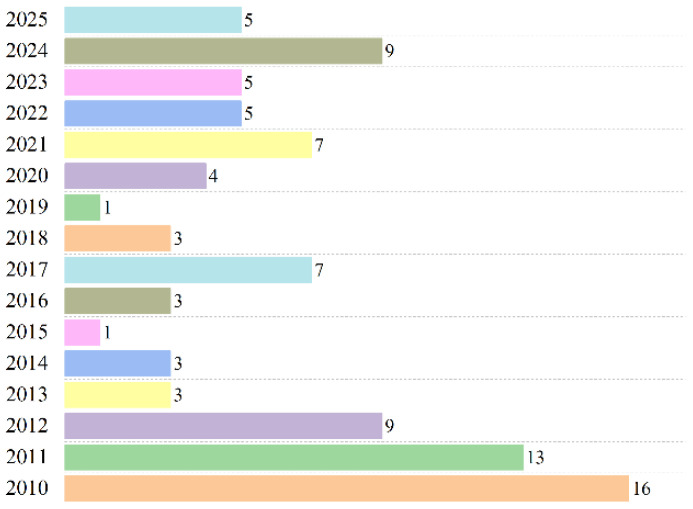
Growth trend of publications on thermoplastic composites in engineering, indicating the rising research interest in recycled and particulate-filled thermoplastic systems over recent years [18].

The introduction of fillers in thermoplastic recycling compensates for property loss due to polymer degradation and offers a route to tailor composite performance for specific structural and semi-structural applications. The impact of these fillers on the characteristics of the composites is influenced by factors such as size, shape, aspect ratio, surface area, and the distribution of fillers throughout the composite [17]. Significant research has focused on thermoplastics combined with particulate fillers, as shown in Figure 3 [18]. Sadik et al. [19] demonstrated the recyclability of the high-density polyethylene (HDPE) and waste glass powder (WGP) composites, showing stable tensile and modulus performance across five reprocessing cycles due to strong filler dispersion and structural integrity.

Similarly, Chaturvedi et al. [20] found that the integration of calcite-rich waste particulates into polyvinyl chloride (PVC) improved tensile strength and dimensional stability while influencing water absorption and plasticisation behaviour, confirming the reinforcing potential of waste particulates in recycled polymers. Adjusting the proportion of fillers in a polymer matrix allows for the tuning of mechanical and sliding wear properties in composite materials [21].

Despite the growing interest in RTCs for civil infrastructure, their structural use remains limited due to inadequate stiffness, tensile, flexural, and impact strength for high-load-bearing applications such as railway sleepers, bridge components, and building elements. For example, composite railway sleepers made from recycled plastics demonstrated durability and environmental resistance but exhibited low tensile and flexural strengths, as well as poor screw-holding capacity, permanent W-shape deflection, and high rail seat deformation under load [22,23]. These shortcomings stem from polymer chain degradation during recycling and the heterogeneity of waste plastic streams, which causes inconsistent behaviour under stress [24,25,26]. While advanced fillers or continuous fibres can improve strength, such solutions often require complex, costly manufacturing, limiting scalability [27,28]. Addressing these gaps requires developing cost-effective, high-strength RTCs with optimised filler systems and rigorously evaluating their mechanical and durability performance under realistic service conditions.

Although interest in thermoplastics recycling is increasing, most prior studies and reviews have either focused on specific thermoplastic–filler systems or fibre-reinforced composites, leaving particulate-filled RTCs relatively underexplored, despite their distinct processing, performance, and sustainability advantages. There is limited comparative analysis of how filler type, particle morphology, loading level, and surface treatment interact with the degraded polymer matrix to influence mechanical, thermal, rheological, and durability properties. Moreover, the scalability of manufacturing methods, the long-term environmental durability of these composites, and the optimisation of filler design parameters for industrial adoption remain insufficiently addressed. Conducting a state-of-the-art review is therefore essential to consolidate existing knowledge, identify consistent trends, critically evaluate limitations, and highlight promising directions for new filler systems and processing innovations. The comprehensive analysis presented in this review provides a strategic knowledge base for researchers and industry, enabling more informed material selection, optimised composite design, and targeted development of next-generation high-performance and sustainable composites from recycled thermoplastics. This review further establishes novel correlations between filler morphology, interfacial behaviour, and environmental performance of particulate-filled RTCs—an integrated synthesis not addressed in previous reviews on recycled or waste-filled polymers.

## 2. Recycled Thermoplastic Composites (RTCs): Overview

### 2.1. Types of Recycled Thermoplastics

**Figure 4:**
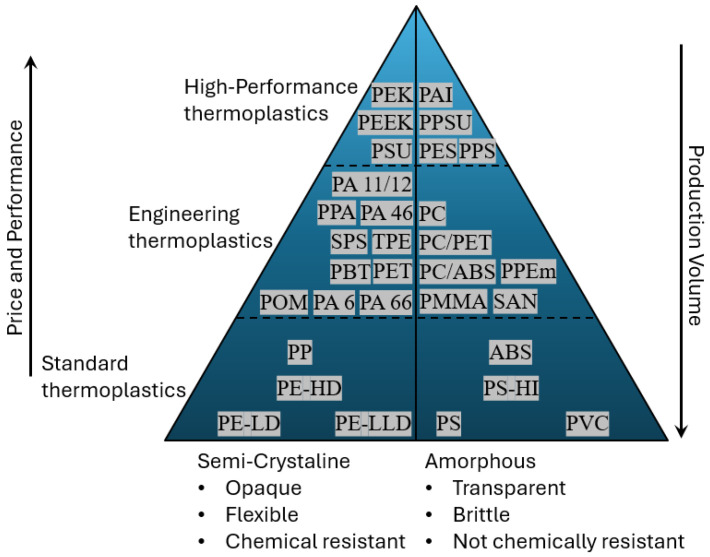
A comparison between standard, engineering, and high-performance thermoplastics [31].

Thermoplastics are composed of linear molecular chains that become soft when heated and harden again upon cooling [29]. Thermoplastic polymers can be categorised into various types based on factors such as the degree of crystallinity, polymerisation method, relative cost for the manufacturing industry, or the volume of consumption [30]. From an engineering design perspective, a classification system that considers the relative cost and the specific applications of the materials holds significant relevance. It is important to note that any classification system is inherently arbitrary [31]. The classification presented in Figure 4 highlights the concept of engineering plastics as materials specifically utilised in technical applications.

Common types of thermoplastic polymers exhibit significantly diverse properties. Semi-crystalline polymers such as polypropylene (PP), low-density polyethylene (LDPE), high-density polyethylene (HDPE), polyethylene terephthalate (PET), polyethylene (PE), polyester polybutylene terephthalate (PBT), polyamide 6 (PA-6), and polyamide imide (PAI) combine structural strength and thermal stability. While amorphous thermoplastics polyvinyl chloride (PVC), polymethyl methacrylate (PMMA), polycarbonate (PC), polystyrene (PS), and acrylonitrile butadiene styrene (ABS), are generally transparent, with their molecules arranged in a random structure [32,33].

These polymers exhibit unique physical, thermal, and electrical properties, making them highly suitable for a diverse range of applications [34,35]. Common thermoplastics include PET, PE, PP, PVC, HDPE, and LDPE, each widely used across packaging, construction, and household products [36]. Although many thermoplastics are recyclable, attention should be directed towards specific types of solid thermoplastic waste, such as PET, HDPE, LDPE, and PP, which represent a substantial proportion [37]. The inherent durability of these materials enables them to maintain their properties even after undergoing multiple recycling processes. The selected parameters—density, modulus, and melting temperature—govern filler compatibility, interfacial bonding, and processing behaviour in particulate-filled recycled thermoplastic composites, and are summarised in Table 1.

This characteristic makes them highly adaptable for a wide range of applications, enhancing their significance in sustainable material management. These thermoplastics represent most commercially utilised polymers, with polyolefins alone comprising 80% of all plastic applications [54].

### 2.2. Classification and Role of Fillers

**Figure 5:**
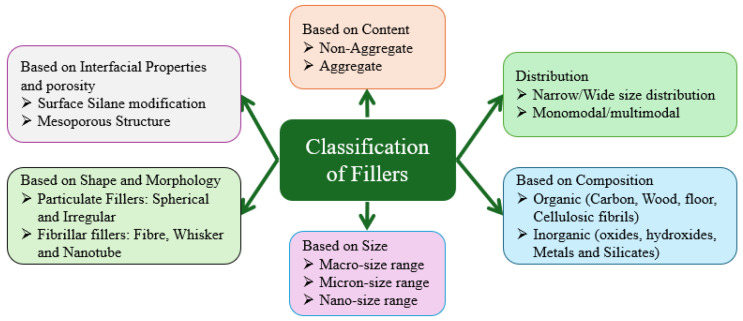
Classification of fillers used in recycled thermoplastic composites (RTCs), based on their origin, morphology, and functional role in modifying composite properties [57].

The classification of fillers in thermoplastic composites extends beyond their chemical origin. Fillers are commonly distinguished based on several critical attributes, including particle size (macro-, micro-, or nano-scale), shape and morphology (e.g., spherical, irregular, fibrous), chemical composition (organic or inorganic), distribution profile (narrow or wide size range; monomodal or multimodal), aggregation state, and surface or interfacial properties such as porosity or surface modification. These parameters significantly affect filler dispersion, interfacial bonding with the polymer matrix, and the resulting mechanical, thermal, and durability performance of the composites [55,56]. Figure 5 illustrates a systematic classification of fillers according to their composition, morphology, and functional contribution to thermoplastic composites [57]. Among the broad classifications, organic fillers, such as wood flour, rice husk, ash, and coconut shell powder, offer advantages including biodegradability and sustainability. In contrast, inorganic fillers like montmorillonite, calcium carbonate (CaCO_3_), silica (SiO_2_), graphite, titanium dioxide (TiO_2_), aluminium oxide (Al_2_O_3_), silicon carbide (SiC), kaolin, zinc oxide (ZnO), magnesium hydroxide (Mg(OH)_2_), boron carbide (B_4_C), carbon powder, and talc, etc., are widely used to improved thermal stability, favourable tribological behaviour, strong interfacial characteristics, and mechanical properties [58].

Numerous studies have investigated the effect of particle size on the mechanical performance of composites. Leidner and Woodhams [59] found that smaller glass beads in polyester composites enhance tensile strength. Gent [60], using Griffith’s fracture criterion, and Needleman [61], through cohesive zone modelling, both concluded that smaller particles require higher stress to debond from the matrix. Similarly, Gent and Park [62] noted that the stress—causing matrix cavitation and debonding increase as particle size decreases. Furthermore, Dubnikova et al. [63] documented a ductile-to-brittle transition in PP composites linked to variations in particle size. In contrast, Mekideche et al. [64] conducted grain size analysis of four silica sands (SS) used in PP-bonded sand composites and found that finer and more uniform grain distributions significantly improved composite performance. Sand types D and B contained up to 60 wt.% fine particles (0.06–0.2 mm), resulting in enhanced flexural strength and stiffness, along with reduced water absorption due to denser packing and reduced porosity. The filler–thermoplastic mechanism, affected by grain size, is based on the physical density and interfacial interaction between sand particles and the molten PP matrix. These findings indicate that finer particle size and well-dispersion enhance load transfer efficiency and delay matrix fracture by strengthening interfacial adhesion and promoting more uniform stress distribution.

### 2.3. Composite Mechanisms: Particulate Filler–Matrix Interaction

**Figure 6:**
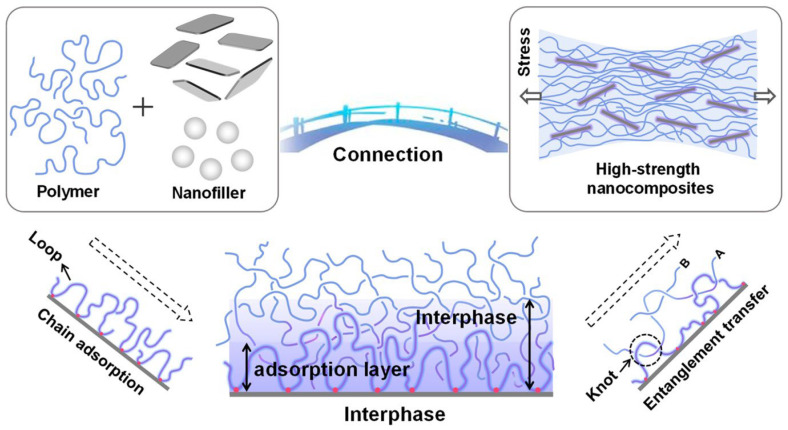
Schematic representation of filler–matrix interaction zones and stress transfer mechanisms in polymer composites, showing polymer chain adsorption, interphase formation, and load transfer across the filler–matrix interface. Chain A represents an adsorbed polymer segment forming a loop structure on the filler surface, Chain B represents a neighbouring polymer chain entangled with Chain A, arrows indicate the direction of stress transfer, and dots/lines denote physical entanglement points and interphase contacts [79].

Particulate fillers are extensively utilised in thermoplastic composites to enhance performance, reduce material costs, and tailor specific properties such as stiffness, thermal conductivity, and dimensional stability. A schematic illustration of the filler–matrix interphase and stress transfer mechanism is presented in Figure 6, highlighting polymer chain adsorption, interphase formation, and load transfer across the filler–matrix interface. Among the most common particulate fillers are talc, calcium carbonate, graphite, clays, silica, carbon black, and nanoparticles, such as graphene. Talc, a platy magnesium silicate, and red mud improve stiffness and heat deflection temperature in polyolefins such as PP [65,66]. Calcium carbonate is widely utilised due to its low cost and ability to enhance dimensional stability and surface finish, particularly in PVC and PP matrices [67,68]. Graphite enhances self-lubrication, thermal conductivity, and wear resistance, particularly in thermoplastics like PP and HDPE. Its effectiveness is contingent on the polymer matrix, filler dispersion, and sample structure [69]. Clays such as montmorillonite and kaolinite have been frequently used as fillers in polymer composites due to their high aspect ratios and ability to form intercalated or exfoliated structures, which significantly enhance flame retardancy and barrier properties. Montmorillonite improves flame resistance by promoting the formation of a char barrier during combustion, thereby slowing heat and mass transfer [70]. Silica is commonly used as a particulate filler in thermoplastic matrices like polyethylene and polystyrene, where it enhances hardness, abrasion resistance, and thermal stability.

Carbon black enhances thermoplastic composites by forming conductive networks that provide electrical conductivity once the percolation threshold is reached. It also offers UV protection by absorbing and dissipating harmful radiation, which helps prevent polymer degradation. These effects render it valuable in automotive, electrical, and packaging applications [71,72,73]. Emerging nanofillers such as graphene and nano-silica are increasingly incorporated into thermoplastic composites due to their capacity to simultaneously enhance mechanical strength, thermal conductivity, and electrical properties, even at low loadings. The performance of particulate-filled thermoplastics is significantly influenced by the quality of filler dispersion and interfacial adhesion with the polymer matrix [74,75]. Some waste thermoplastic composites, such as LDPE and HDPE, involve sand particles acting as a reinforcing agent, enhancing structural integrity by providing a stable framework within the polymer matrix. This interaction reduces thermal expansion, improves thermal conductivity, and restricts polymer chain mobility, thereby enhancing dimensional stability and resistance to deformation [76]. Moreover, sand filler in waste PET plastic composites minimises water absorption and increases fire resistance by filling voids and creating a cohesive structure through mechanical interlocking and partial wetting [77].

Overall, particulate fillers play an essential role in modifying the structural and functional characteristics of thermoplastic composites without the complexities introduced by fibre-matrix alignment or processing issues associated with fibrous reinforcements. Abou-Kandil et al. [78] observed through SEM examination that the morphological characteristics of HDPE nanocomposites were strongly influenced by the size and content of ZnO nanoparticles. At 2.5 wt.% ZnO with particles calcinated at 350 °C (Z4, ~25 nm), the nanoparticles were uniformly dispersed with minimal agglomeration, resulting in a smooth fracture surface and enhanced mechanical integrity. However, larger particle sizes or higher filler contents led to visible agglomeration and void formation. The filler–thermoplastic mechanism influencing morphology was determined by nanoparticle surface treatment and dispersion quality, which controlled interfacial bonding and composite homogeneity.

## 3. Effect of Fillers on the Manufacturing Process of RTCs

The incorporation of fillers into recycled thermoplastics can influence their processability during manufacturing. A comparative summary of major manufacturing methods, their advantages, limitations, and resulting composite properties is presented in Table 2. Fillers alter the melt rheology, heat transfer characteristics, and shrinkage behaviour of polymer melts, which in turn affect mould filling, cycle time, and dimensional stability of the final product [80]. In extrusion and extrusion blow moulding, for example, the addition of mineral fillers such as talc to recycled polyethylene (rPE) enhances crystallinity and tensile strength but increases melt viscosity, reducing flowability and requiring adjustments to screw design and temperature profiles [11]. Recent research has explored compression moulding, extrusion, injection moulding, and additive manufacturing to process post-consumer and post-industrial plastics efficiently and competitively with virgin polymers [81,82,83].

Polymer matrix composites are being manufactured using compression or injection moulding in a cost-effective way [84,85]. Kumi-Larbi Jnr et al. [86] investigated recycled plastic-bonded natural sand composites through two processing methods: the oven moulding technique (OMT) and the heat-mixing technique (HMT). In OMT, sand and thermoplastic (LDPE or HDPE) mixtures were layered and heated in an oven at 250–375 °C and thereafter mixed and cast into preheated moulds. In HMT, the thermoplastic was melted on a hot plate, mixed with sand, and then moulded. The processing temperature and time significantly affected the final mechanical properties: optimal strengths were achieved between 250 °C and 325 °C, whereas higher temperatures caused thermal degradation and reduced strength. The authors noted that lower temperatures resulted in heterogeneous composites due to poor mixing. Sadik et al. [19] processed HDPE and WGP composites using melt compounding on a two-roll mill, followed by compression moulding. The materials were thermally compounded at 150 °C for 12 min using a Prep-Mill two-roll mill, where HDPE was first melted, WGP was gradually added, and the blend was then homogenised. The composite paste was shaped into sheets using a hydraulic hot press at 160 ± 10 °C and 7.355 MPa pressure for 10 min. The processing parameters significantly impacted the dispersion, interfacial adhesion, and mechanical integrity of the composites. The final properties depended heavily on appropriate mixing time, temperature, and pressure to achieve uniform filler distribution and minimise agglomeration.

Furthermore, Periasamy et al. [87] demonstrated that magnetic wave-assisted injection moulding enhanced filler dispersion and interfacial bonding in HDPE composites reinforced with titanium dioxide (TiO_2_f) and alumina (Al_2_O_3_f) bio-ceramic fillers, leading to improved mechanical performance. Similarly, Babatunde et al. [77] processed PET–sand composites through direct melting and manual mixing, confirming that adequate blending and temperature control are essential to achieve homogeneous filler distribution and strong particle cohesion. Zahran [88] employed compression moulding to fabricate sand-reinforced LDPE composites and highlighted that the absence of flow-induced forces limited mechanical interlocking, making interfacial adhesion a dominant factor in composite strength. Jeyachandran et al. [89] further reported that melt compounding, extrusion, and fused filament fabrication (FFF) of HDPE–bioactive glass composites produced uniform filler dispersion and robust interlayer bonding, resulting in superior tensile and flexural properties. Moreover, followed by injection moulding into 4 mm-thick plates, Abou-Kandil et al. [78] processed ZnO/HDPE nanocomposites by first melt mixing molten HDPE pellets with treated ZnO nanoparticles using a twin-screw extruder. The authors demonstrated that the calcination temperature of ZnO nanoparticles greatly influenced the composite properties. Nanoparticles calcinated at 350 °C (sample Z4, ~25 nm) yielded optimal outcomes for tensile strength, hardness, and UV shielding, due to uniform dispersion and ideal particle size. In contrast, higher calcination temperatures (≥400 °C) produced larger particles prone to agglomeration and void formation, which degraded mechanical performance and visible light transparency. The filler–thermoplastic mechanism was influenced by the calcination-induced particle morphology and its effect on dispersion quality and interfacial bonding within the HDPE matrix.

**Table 2:** Comparative summary of manufacturing techniques for particulate-filled RTCs, with associated advantages, limitations, and property outcomes.

Matrix Type	Filler Type	Manufacturing Method	Advantages	Limitations	Typical Property Outcomes	Refs.
LDPE/HDPE	Silica sand	Oven Moulding (OMT), Heat Mixing (HMT)	Simple, low-cost processing; suitable for large parts	Poor filler dispersion at low temperature; thermal degradation > 325 °C	Flexural modulus ranged between 0.5 and 0.7 GPa; improved density and stiffness	[86]
HDPE	WGP	Melt compounding (two-roll mill) + compression moulding	Good filler distribution; improved stiffness	Increased viscosity and reduced ductility	Modulus ↑ >2x; tensile strength maintained up to 25 MPa with compatibiliser.	[19]
HDPE	TiO_2f_, Al_2_O_3f_	Magnetic wave-assisted injection moulding	Enhanced filler dispersion; strong interfacial bonding	Requires complex setup and magnetic control	Flexural/tensile strength ↑37%/122%; improved surface uniformity.	[87]
PET	Sand	Direct melting and manual mixing	Uses 100% recycled PET; simple processing	Manual blending causes nonuniform dispersion	Flexural strength ↑ to 2.55 MPa at 1:3 PET:sand ratio.	[77]
LDPE	Sand	Compression moulding	Economical; no high-pressure flow	Limited mechanical interlocking	Moderate tensile strength (8–14 MPa); improved stability.	[88]
HDPE	BAG	Melt compounding + extrusion + FFF printing	Excellent control of porosity; strong interlayer bonding	High printing cost, slower productivity	Tensile modulus ↑ 56%; flexural modulus ↑40%.	[89]
HDPE	ZnO	Twin-screw extrusion + injection moulding	Enhanced dispersion; UV stability improvement	Agglomeration at > 2.5 wt.% filler	Tensile strength ↑19%; hardness ↑15%; improved UV resistance.	[78]
rLDPE/LLDPE	Talc	Twin-screw extrusion + blow moulding	High production rate; improved crystallinity	Increased melt viscosity	Improved stiffness and heat resistance; slight strength loss	[11]
HDPE	BAG	Dual-head FFF 3D printing	High-resolution fabrication; controlled filler orientation	Limited scalability	Uniform microstructure; tensile/flexural strength ↓5–13%/↑15–25%.	[89]

Note: ↑—increase,↓—decrease.

## 4. Properties of Particulate-Filled RTCs

### 4.1. Physical Properties of Particulate-Filled RTCs

#### 4.1.1. Morphological and Density Properties

**Figure 7:**
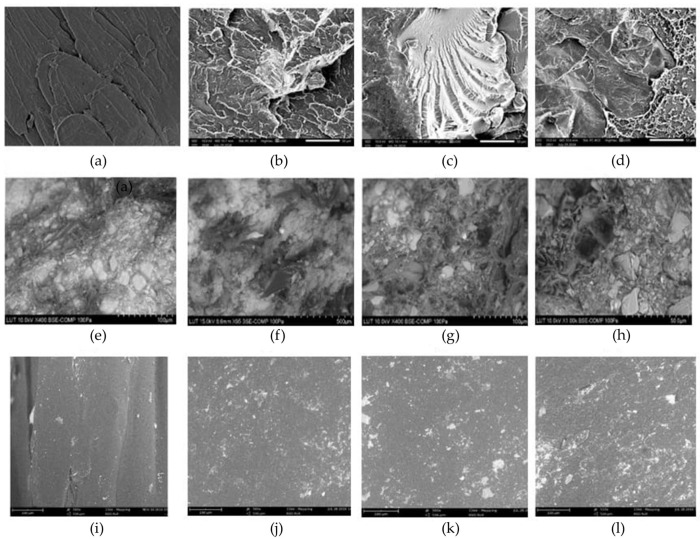
SEM micrographs showing the morphological characteristics and filler–matrix interactions in RTCs: (**a**–**d**) fractured surfaces of PET-based composites—(**a**) bPET, (**b**) bPET_EGs, (**c**) bPET_EGm, and (**d**) bPET_EGb; (**e**–**h**) recycled HDPE composites with mineral fillers—(**e**) GYP40, (**f**) GYP60, (**g**) SS40, and (**h**) SS60; (**i**–**l**) rPP composites with periwinkle shell (PS) particles of varying sizes—(**i**) rPP, (**j**) 150 µm, (**k**) 300 µm, and (**l**) 425 µm [83,93,94].

**Figure 8:**
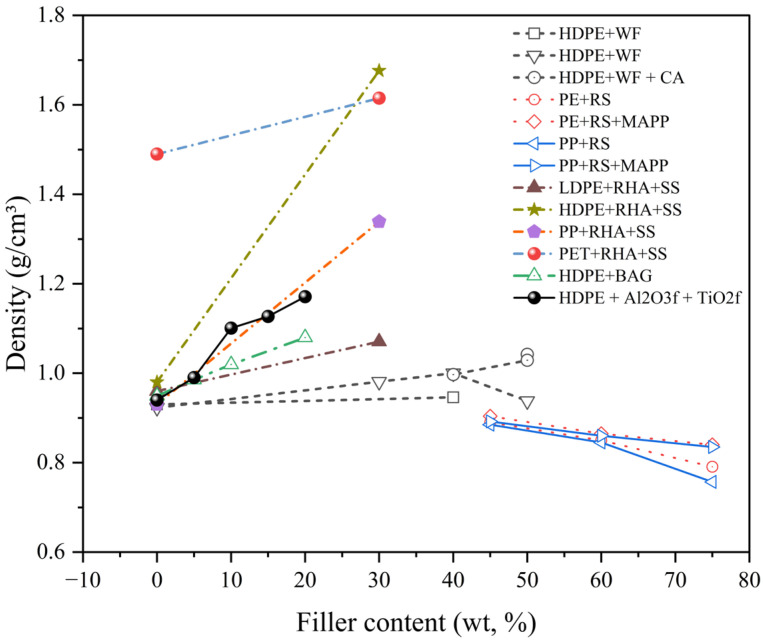
Effect of filler content on the density of various RTCs, showing the densification trend with increasing inorganic filler incorporation [87,89,90,91,92].

Physical properties of particulate-filled RTCs encompass characteristics such as density, porosity, and dimensional stability, which influence both processing and end-use performance. The incorporation of fillers can increase the composite’s density, reduce shrinkage, and enhance structural uniformity, although excessive filler loading may lead to agglomeration and compromised homogeneity, as shown in Figure 7. Figure 8 illustrates the influence of filler content on the density of various particulate-filled thermoplastic composites. For virgin HDPE and recycled HDPE (rHDPE) filled with wood flour (WF), density increased slightly with filler content due to the low density of the organic filler. However, when compatibilisers such as coupling agents (CA) were introduced, the trend became more pronounced, reflecting improved packing and reduced voids [90].

In contrast, rice straw (RS)-filled PE and PP composites exhibited a gradual decrease in density with increasing filler content, particularly without maleic anhydride polypropylene (MAPP), likely due to the lower intrinsic density of RS and increased porosity [91]. Significantly, rLDPE, rHDPE, rPP, and rPET filled with 15% rice husk ash (RHA) and 15% silica sand (600 μm) composites displayed a sharp increase in density, ranging from ~1.1 g/cm^3^ to above 1.6 g/cm^3^, highlighting the dense nature of the inorganic fillers and their superior interfacial packing compared to polymer matrices [92].

Similarly, the addition of bioactive glass (BAG) to virgin HDPE led to a consistent rise in density from 0.95 g/cm^3^ at 0 wt.% to 1.08 g/cm^3^ at 20 wt.%, reflecting the densifying effect of mineral-based fillers and the improved matrix–filler adhesion enabled by BAG’s surface chemistry [89]. Additionally, Tufa et al. [95] found that the density of LDPE-HDPE sand-plastic composites ranged from 0.89 to 1.4 g/cm^3^, with the highest density recorded at 22.2 wt.% LDPE, 22.8 wt.% HDPE, and 45 wt.% sand. Lower density (0.89 g/cm^3^) occurred in samples with 60 wt.% LDPE and 30 wt.% 0.5 mm sand. Variations were due to filler dispersion and porosity from non-uniform mixing.

Inorganic fillers such as RHA, sand, and BAG significantly increased density with higher filler loading due to their higher specific gravity and improved particle packing, whereas wood-flour-based systems showed marginal changes. Enhanced interfacial compaction in HDPE + RHA + SS and HDPE + Al_2_O_3f_ + TiO_2f_ systems contributed to superior density stability.

#### 4.1.2. Hardness Properties

**Figure 9:**
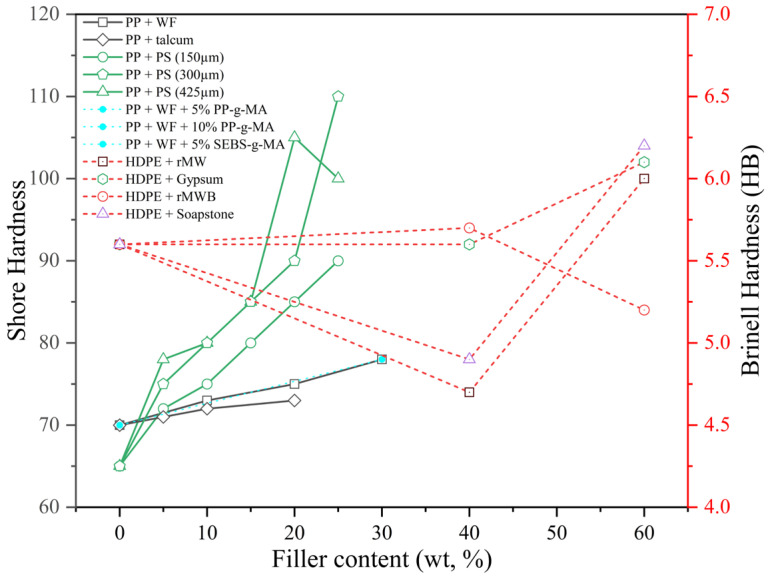
Effect of filler content on the hardness of various RTCs, demonstrating the progressive increase in surface hardness with higher filler loading across different matrices [76,83,94].

The hardness of recycled thermoplastic composites demonstrated a strong dependence on both filler loading and particle size. As illustrated in Figure 9, the shore hardness of Periwinkle Shell (PS)-PP composites increased progressively with filler content for all particle sizes (150 µm, 300 µm, and 425 µm), reaching peak values at 25–30 wt.%. For instance, composites with 300 µm particles exhibited a marked increase in surface resistance from 65 (Shore) at 0 wt.% to 110 at 30 wt.%, representing a ~69% improvement.

Similarly, a larger particle of periwinkle shell (PS_425 µm) in rPP reached comparable hardness (105 Shore) at 30 wt.%, likely due to enhanced packing and increased contact area, though with more fluctuation. In contrast, smaller particles PS (150 µm) in rPP yielded a more moderate increase (from 65 to 95 Shore), suggesting a trade-off between dispersion and filler–matrix friction [94]. Sormunen and Kärki [83] reported that Brinell hardness (HB) values also showed variable responses across different recycled HDPE-based composites. For example, soapstone-filled HDPE showed the highest hardness (6.1 HB), surpassing the reference HDPE (5.6 HB). Gypsum- and recycled mineral wool (rMW)-filled composites also exhibited improved hardness at 60 wt.% filler. In wood flour (WF)-filled PP composites, Shore hardness steadily increased with filler content, from 70 (neat PP) to 78 at 30 vol%, both with and without compatibilisers. Compatibiliser addition (PP-g-MA or SEBS-g-MA) helped maintain or slightly enhance hardness even after water immersion. Meanwhile, talcum-filled PP showed a more modest increase from 71 to 73 Shore (at 20 vol%), reflecting the densifying but softer nature of talc compared to mineral or fibre reinforcements [76].

These results affirm that filler type, content, particle morphology, and interfacial compatibility are critical factors influencing the surface hardness of RTCs, with mineral fillers generally outperforming lignocellulosic reinforcements in enhancing indentation resistance. Moreover, Grigorescu et al. [96] investigated the physical behaviour of recycled polypropylene (rPP) composites reinforced with waste printed circuit board (WPCB) powder, focusing on crystallinity and modulus changes. X-ray Diffraction (XRD) analysis showed a reduction in crystallinity from 59.6% in neat rPP to 44.8% in rPP with 30% WPCB and further down to 38.2% with additional SBS elastomer. This reduction is attributed to filler-induced disruption of the rPP crystal lattice and interfacial incompatibility (Figure 7). However, Dynamic Mechanical Analysis (DMA) results confirmed a 35–42% increase in storage modulus at 30 °C for WPCB-filled composites, indicating improved stiffness due to the rigid glass fibre content in the filler. Thus, despite lower crystallinity, the particulate WPCB physically strengthened the rPP matrix by mechanical interlocking and constrained chain mobility.

Similarly, Adhikari et al. [97] found that rHDPE composites with pine dust showed improved ultimate strength up to 20 wt.% at 250–500 μm particle size. Beyond this, strength declined due to filler agglomeration and weak interfacial adhesion. Smaller particles (<250 μm) led to clustering and reduced strength. Optimal physical properties were linked to better filler dispersion and matrix interaction. Psyanchin et al. [98] reported that incorporating 13 wt.% aluminosilicate microspheres into rPP increased processing torque and reduced melt flow, indicating higher melt viscosity. However, modifying the composite with 0.5–1 wt.% stearic acid improved particle dispersion and decreased torque, resulting in the melt flow index rising from 12.4 to 30.6 g/10 min. The physical improvements were attributed to reduced filler agglomeration and enhanced interface lubrication between the filler and rPP matrix. Kuciel et al. [93] found that bioPET (bPET) composites filled with 10 wt.% eggshell (EG) exhibited particle-size-dependent physical behaviour. Finer particles (EGs~67 µm) reduced maximum displacement from 27.7 mm to 9.1 mm, indicating enhanced rigidity and structural integrity. In contrast, larger particles (EGb~747 µm) led to poorer filler–matrix interaction and higher deformation. Improved dispersion and interfacial contact of fine particles were key to the enhanced physical performance (Figure 7). The SEM analysis of SS40 and SS60 revealed porosity and dislocation of soapstone particles from the matrix—more pronounced in SS60—indicating cavity formation that contributed to the high variability in its tensile strength, while GYP40 and GYP60 showed well-dispersed gypsum particulates with clean surfaces, suggesting weak matrix adhesion but improved uniformity in GYP60 [83].

The results showed contrasting results on the density and hardness with filler content, indicating that further investigation is needed to determine the optimal content of fillers to enhance the physical properties of thermoplastics.

Shore and Brinell hardness values increased with higher filler loading due to enhanced stiffness and surface compaction, particularly in PP + PS and HDPE + mineral systems. Fine particle sizes and compatibilised interfaces (PP-g-MA, SEBS-g-MA) contributed to improved filler–matrix adhesion and hardness stability.

### 4.2. Mechanical Properties of Particulate-Filled RTCs

The incorporation of particulate fillers into recycled thermoplastic composites can modify mechanical performance, including stiffness, strength, and toughness, depending on the filler type, size, shape, surface treatment, and loading percentage. Targeted optimisation of these parameters has been the focus of numerous studies aiming to overcome the inherent property limitations of recycled thermoplastic matrices. Table 3 consolidates representative findings from recent literature, summarising matrix–filler combinations, particle characteristics, loading levels, study objectives, and the resulting changes in mechanical and tribological performance. This synthesis provides a comparative perspective on filler-induced property modifications, serving as a basis for the detailed discussion of tensile, flexural, and impact behaviour in the following sections.

#### 4.2.1. Tensile Strength

**Figure 10:**
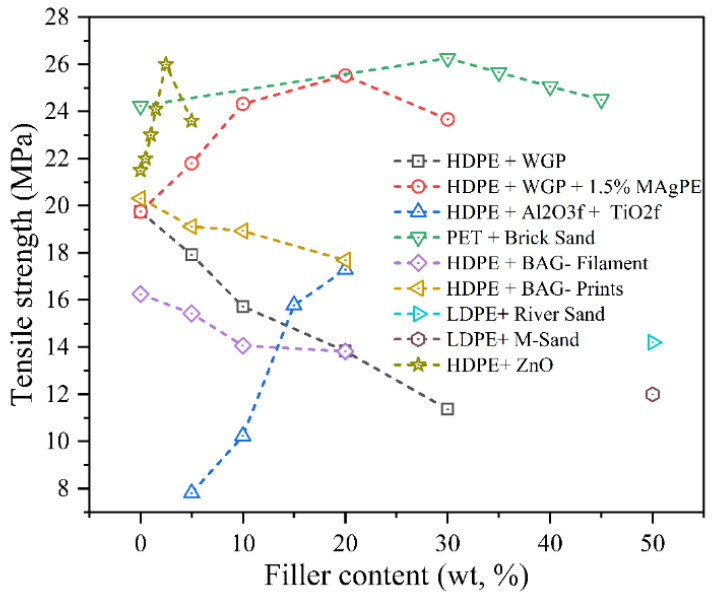
Effect of filler content on the tensile strength of various RTCs, illustrating the influence of filler loading and interfacial bonding on strength enhancement or reduction across different matrices [19,78,87,89,100,101].

The effect of particulate fillers on tensile strength depends on factors such as filler content, particle-matrix adhesion, dispersion quality, and the inherent strength of both the matrix and filler materials, as presented in Figure 10. Sadik et al. [19] investigated the influence of varying waste glass powder content (particle size 20–60 nm) on the tensile behaviour of recycled HDPE (rHDPE). As the WGP content increased from 0 to 20 wt.%, the tensile strength declined markedly from 19.75 MPa to 13.83 MPa (−30%), the tensile modulus dropped from 690 to 602.8 MPa (−13%), and the elongation at break plummeted from 707.3% to 21%. These pronounced reductions were attributed to the rigid, non-deformable nature of glass particles and their poor interfacial adhesion with the polymer matrix, which led to particle agglomeration and the formation of stress concentration zones that hindered effective load transfer, particularly beyond the critical filler volume fraction where polymer chain mobility becomes restricted and ductility sharply declines. For context, fibre-reinforced recycled thermoplastics (rHDPE with 0–20 wt.% banana fibre) show tensile strength decreasing from 19 MPa to 11.9 MPa (−42%) and elastic modulus reducing from 1.2 GPa to 0.3 GPa (−77%) [99], demonstrating that untreated fibre systems suffer greater degradation in ductility and stiffness than particulate-filled RTCs. However, incorporating 1.5 wt.% maleic anhydride-grafted polyethylene (MAgPE) into rHDPE/WGP composites significantly enhanced interfacial bonding and compatibility. As a result, the tensile strength improved from 19.75 MPa (neat HDPE) to 25.53 MPa at 20 wt.% WGP, while stiffness increased markedly from 690 MPa to 1564.81 MPa. Although the elongation at break decreased from 707% to 16.84%, this reduction reflects the typical stiffness–ductility trade-off observed in filled thermoplastics. Even at higher WGP loadings (30 wt.%), tensile strength was maintained at 23.67 MPa, confirming the effectiveness of compatibilisation in preserving mechanical performance [19]. Additionally, Periasamy et al. [87] investigated the tensile strength of HDPE composites reinforced with constant 5 vol% titanium dioxide (TiO_2_f) and varying alumina (Al_2_O_3_f) content (0–15 vol%) using magnetic wave-assisted injection moulding. The tensile strength increased progressively from 7.81 ± 0.23 MPa (with only TiO_2_f) to a maximum of 17.29 ± 0.52 MPa at 15 vol% Al_2_O_3_f, indicating a 1.21-fold improvement. This enhancement is attributed to the improved filler-matrix interfacial bonding and homogeneous filler distribution achieved by the magnetic action. The increase in tensile strength was driven by the ceramic fillers’ ability to resist particle movement under tensile stress, while decreases in strength were not observed within the tested range, confirming a positive correlation between alumina content and tensile reinforcement in HDPE composites.

Moulai Arbi et al. [100] investigated the mechanical performance of PET reinforced with brick sand and found that both tensile strength and Young’s modulus peaked at 30% brick sand content, increasing from 25.45 MPa and 2700 MPa to 26.26 MPa and 2771 MPa, respectively. These enhancements were attributed to the effective dispersion of filler particles and strong interfacial adhesion, which facilitated stress transfer and improved stiffness. Beyond 30% filler content, the agglomeration of brick particles disrupted uniform dispersion, weakened matrix–filler bonding, and led to material inhomogeneity, resulting in declines in both tensile strength and modulus. The filler–thermoplastic mechanism influencing these properties was governed by the quality of particle dispersion and the distribution of reinforcement within the PET matrix. In contrast, Jeyachandran et al. [89] investigated the tensile strength of HDPE composites reinforced with bioactive glass (BAG) fabricated via fused filament fabrication (FFF). The tensile modulus of printed composites increased linearly with BAG content, reaching 1230.32 MPa for 20 wt.% BAG, which was 1.82 times higher than neat HDPE. However, the ultimate tensile strength (UTS) decreased from 20.31 MPa for neat HDPE to 17.69 MPa for 20 wt.% BAG due to reduced matrix deformability and stress concentration at the filler–matrix interface. The filler–thermoplastic mechanism influencing tensile strength involved homogeneous BAG dispersion and interfacial bonding, which improved stiffness but introduced brittleness with increasing filler loading.

Mohan et al. [101] examined the split tensile strength of recycled low-density polyethylene (rLDPE) composites filled with river sand and manufactured sand. The highest average tensile strength was recorded for river sand composites at 14.2 MPa, compared to 12.0 MPa for manufactured sand. This improvement was attributed to better particle packing and reduced porosity in river sand mixtures, enhancing stress distribution. The filler–thermoplastic mechanism influencing tensile strength was governed by the uniform dispersion of sand particles and the bonding integrity within the heated LDPE matrix, enabling effective load transfer under tensile stress. Abou-Kandil et al. [78] studied the mechanical behaviour of HDPE nanocomposites reinforced with zinc oxide (ZnO) nanoparticles and found that tensile strength increased with ZnO content up to 2.5 wt.%, reaching a maximum of 26.0 MPa with particles calcinated at 350 °C (Z4, ~25 nm), compared to 21.8 MPa for the unfilled matrix, approximately 19%. Elongation at break decreased progressively with increasing ZnO content and particle size. The enhancement in tensile strength was attributed to homogeneous nanoparticle dispersion and strong interfacial bonding, which facilitated stress transfer and reinforcement, while the reduction in elongation was linked to increased crystallinity and restricted chain mobility. The filler–thermoplastic mechanism governing both properties was driven by the balance between effective dispersion and agglomeration, where excessive filler content or poor dispersion led to void formation, brittle fracture, and reduced mechanical flexibility.

The tensile behaviour of particulate-filled RTCs strongly reflects the balance between filler rigidity and interfacial bonding quality. Inorganic fillers such as glass powder, alumina, TiO_2_, and sand generally impart higher stiffness and tensile modulus due to their rigid crystalline structure and strong load–transfer efficiency. Their thermal stability and dimensional rigidity enhance stress resistance but simultaneously restrict polymer chain mobility, causing moderate reductions in elongation at break. Conversely, bio-fillers or weakly bonded fillers often exhibit lower tensile reinforcement because their hydrophilic surfaces promote poor wetting, microvoid formation, and early crack initiation under stress. Nano-scale fillers like ZnO and Al_2_O_3_ achieve superior reinforcement even at low loadings due to their large surface area and homogeneous dispersion, which improve interfacial stress transfer. Overall, the results demonstrate that tensile strength enhancement in RTCs is not solely governed by filler content but primarily by particle–matrix adhesion, morphology, and compatibility—underscoring the importance of compatibilisers and surface treatments in achieving high-strength, durable composites.

#### 4.2.2. Flexural Strength

**Figure 11:**
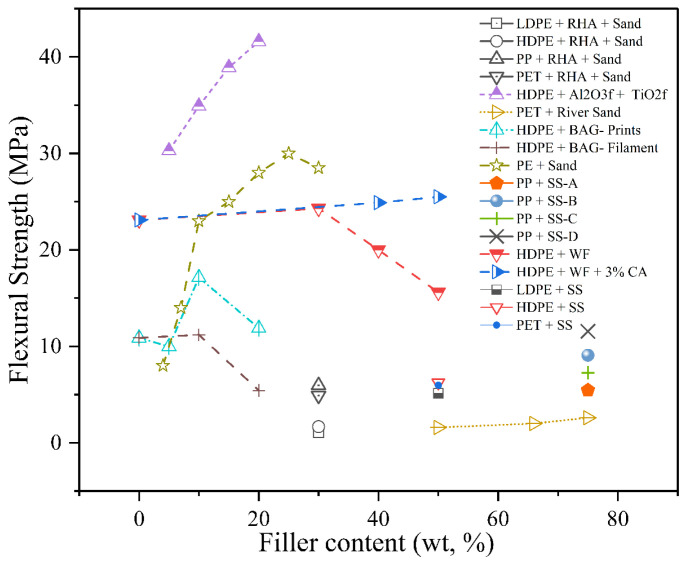
Effect of filler content on the flexural strength of various RTCs, highlighting how filler type, dispersion quality, and loading level influence flexural reinforcement and stiffness improvement across different polymer matrices [64,77,87,89,90,92,102,103].

The incorporation of particulate fillers can improve flexural strength by increasing stiffness and load-bearing capacity, though excessive or poorly bonded fillers may lead to brittleness and premature failure, as displayed in Figure 11. Periasamy et al. [87] investigated the effect of TiO_2_ and Al_2_O_3_ bio-ceramic fillers on the flexural behaviour of HDPE composites fabricated using a magnetically assisted injection moulding process. With a constant 5 vol% TiO_2_ and increasing Al_2_O_3_ content from 0 to 15 vol%, the flexural strength improved significantly—from 30.28 MPa (unfilled HDPE+TiO_2_) to a maximum of 41.61 MPa at 15 vol% Al_2_O_3_. The authors attributed this 37.4% increase to the formation of a strong interfacial bond and uniform filler dispersion enabled by magnetic field processing. Similarly, a progressive enhancement in flexural properties was observed with increasing filler content in HDPE–BAG composites, as reported by Jeyachandran et al. [89]. The neat HDPE printed sample exhibited a flexural strength of 22.87 ± 0.34 MPa and a modulus of 774.24 ± 11.20 MPa. Upon the addition of 5 wt.% filler, the strength increased slightly to 23.90 ± 0.33 MPa, accompanied by a 22% rise in modulus. A more substantial improvement was recorded at 10 wt.%, where flexural strength and modulus reached 25.74 ± 0.30 MPa and 1050 ± 13.11 MPa, respectively. The maximum values were achieved at 20 wt.% filler, yielding 27.53 ± 0.32 MPa strength and 1121 ± 15.12 MPa modulus. These improvements were attributed to the rigid nature of BAG, enhanced filler–matrix interfacial adhesion, and improved dispersion.

Adhikary et al. [90] investigated the flexural behaviour of wood–plastic composites (HDPE-WF) fabricated from both recycled and virgin high-density polyethylene reinforced with 30–50 wt.% Pinus radiata wood flour, with and without coupling agents. The flexural strength of rHDPE-based composites ranged from 15.6 to 25.5 MPa, while the flexural modulus (MOE) increased from 1.3 to 1.97 GPa. In contrast, HDPE-based composites exhibited lower flexural strength values, ranging from 14.4 to 17.9 MPa, and modulus values from 1.06 to 1.34 GPa, highlighting the superior reinforcing effect of recycled HDPE. Notably, the incorporation of 3–5 wt.% maleated polypropylene (MAPP) into rHDPE–wood composites significantly improved both flexural strength and modulus, reaching 25.5 MPa and 1.88 GPa, respectively, at 50 wt.% wood flour. These enhancements were attributed to improved interfacial bonding via esterification between MAPP and the hydroxyl groups of wood. Mekideche et al. [64] investigated PP composites prepared using four silica sand types (SS-A to SS-D), each containing 75 wt.% filler and 25 wt.% recycled PP, where flexural strength ranged from 5.45 to 11.56 MPa and modulus from 1061 to 1557 MPa, indicating that mineralogical compatibility and particle morphology strongly influence mechanical outcomes.

In a study, Soni et al. [92] examined recycled thermoplastic composites containing 15 wt.% rice husk ash and 15 wt.% natural sand. The PP- RHA- Sand system achieved the highest flexural strength (5.96 MPa), followed by PET- RHA- Sand (4.90 MPa), while HDPE and LDPE composites exhibited significantly lower strengths (1.68 and 1.07 MPa, respectively). These variations were attributed to the intrinsic stiffness of the matrix polymers and the degree of filler encapsulation and bonding. A positive correlation between density and strength was also observed, highlighting the importance of efficient matrix–filler packing. In another sand-based system, Babatunde et al. [77] evaluated waste PET–river sand composites at different mix ratios (1:1, 1:2, 1:3) and found that flexural strength increased from 1.55 MPa to 2.55 MPa as the sand content increased, confirming the reinforcing effect of sand particles. Despite high filler loading, sufficient PET binder maintained particle cohesion, allowing the composite to exceed the BS 5628-1:1992 minimum flexural strength requirement for mortar bricks.

These findings support the concept that optimised filler-to-binder ratios can produce structurally viable recycled materials for non-load-bearing and semi-structural applications. Yi et al. [102] explored the development of structural composites using sand as filler and PE as a thermoplastic binder, targeting ultra-low binder content (4–30 wt.%) through a compaction self-assembly (CSA) method. At just 4 wt.%, the PE–sand composite achieved a flexural strength of ~8 MPa, which increased to ~14 MPa at 7 wt.% and peaked at ~30 MPa with 25 wt.% PE. The most significant gain occurred between 7 and 10 wt.%, where the strength rose from ~14 to 23 MPa, indicating efficient load transfer due to binder micro-agglomeration and interfacial capillary forces. While the HDPE–SS composite (50 wt.% HDPE + 50 wt.% silica sand) exhibited the highest flexural strength of 6.24 MPa, followed by the PET–SS composite (50 wt.% LDPE + 20 wt.% PET + 30 wt.% sand) with 5.96 MPa, and the LDPE–SS composite (50 wt.% LDPE + 50 wt.% sand) with 5.13 MPa. This trend indicates that HDPE, due to its higher modulus and better interfacial bonding with sand particles, contributes more effectively to flexural load resistance than LDPE or PET [103].

The flexural performance of particulate-filled RTCs depends on filler rigidity, particle geometry, and interfacial bonding strength. Inorganic fillers such as sand, alumina, and bioactive glass offer superior stiffness and load-bearing capacity due to their high elastic modulus and efficient stress transfer across the filler–matrix interface. Their rigid crystalline structure and dimensional stability promote bending resistance but can increase brittleness when dispersion is poor. In contrast, bio-fillers such as rice husk ash or wood flour exhibit lower flexural reinforcement because of their porous morphology, irregular particle shape, and hydrophilic surfaces, which weaken interfacial adhesion and promote microcrack initiation. Compatibilisers like MAPP or MAgPE effectively mitigate these issues by improving surface polarity and enabling better stress distribution. Moreover, higher-modulus matrices such as HDPE or PP exhibit stronger reinforcement responses compared to LDPE or PET, as they better complement the stiffness of mineral fillers. Overall, the comparative studies confirm that the synergy between filler type, matrix modulus, and interfacial adhesion governs the bending strength and stiffness of RTCs.

#### 4.2.3. Impact Strength

**Figure 12:**
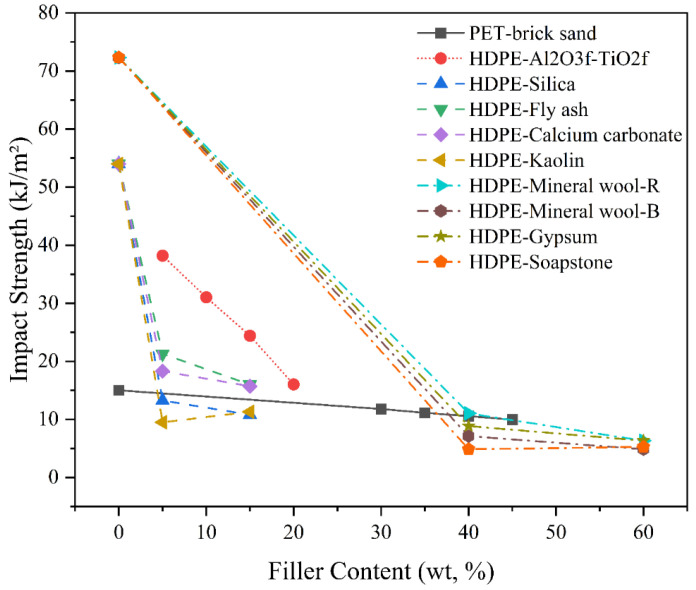
Effect of filler content on the impact strength of different RTCs, highlighting the reduction in impact resistance at higher filler loadings and the role of filler–matrix compatibility in energy absorption behaviour [83,87,100,104].

The addition of particulate fillers can either enhance or reduce impact strength depending on factors such as filler type, size, dispersion, matrix ductility, filler morphology, and interfacial bonding quality, as demonstrated across several recent studies, as shown in Figure 12. Kumi-Larbi Jnr et al. [86] discovered that HDPE-bonded sand composites exhibited higher impact-related toughness (1.0–2.1 MJ/m^3^) compared to LDPE (0.6–2.3 MJ/m^3^), with both achieving optimal performance at around 67–75 wt.% sand addition depending on particle size. Higher sand content and finer particles enhanced energy absorption by improving matrix–particle interlocking. However, increasing processing temperatures beyond 325 °C led to thermal degradation of the thermoplastic, significantly reducing the work of fracture and thus the impact resistance to embrittlement of the binder matrix. Periasamy et al. [87] observed that the impact strength of HDPE composites decreased with increasing alumina (Al_2_O_3_f) filler content, despite the constant inclusion of 5 vol% titanium dioxide (TiO_2_f). The highest impact strength was 38.19 ± 0.88 kJ/m^2^ for the composites without Al_2_O_3_f, which decreased to 16.01 ± 0.37 kJ/m^2^ at 15 vol% Al_2_O_3_f. The reduction in impact strength was attributed to the increased brittleness introduced by higher ceramic filler content and the inability of TiO_2_f to absorb high-impact loads, leading to surface fracture initiation in the HDPE matrix.

This embrittling effect of rigid fillers was similarly reflected in Moulai Arbi et al. [100] found that the impact strength of PET-brick sand composites decreased progressively with increasing brick sand content, dropping from 15 kJ/m^2^ for pure PET to 9.95 kJ/m^2^ at 45% filler content. The decline in impact resistance was linked to poor dispersion and the formation of filler agglomerates, which acted as stress concentrators and weakened the matrix. The filler–thermoplastic mechanism influencing impact behaviour was dominated by interfacial adhesion and morphological uniformity between brick particles and the PET matrix. In line with this, Rasib et al. [104] reported a substantial reduction in notched Izod impact strength of HDPE filled with various industrial waste fillers—including silica, kaolin, CaCO_3_, and fly ash, with neat HDPE exhibiting the highest value (54.0 ± 9.2 kJ/m^2^). Among the filled systems, HDPE with 5 wt.% fly ash retained the highest impact strength (21.6 ± 6.3 kJ/m^2^), while HDPE/kaolin composites showed the poorest performance (9.5 ± 1.3 kJ/m^2^). The reduction in impact resistance was attributed to weak filler–matrix adhesion, increased stress concentration sites, and filler agglomeration, which accelerated crack initiation and propagation.

In contrast to these reductions, Petri Sormunen et al. [83] demonstrated that the incorporation of recycled mineral fillers into HDPE composites resulted in a significant reduction in impact strength—up to 90% lower than the neat HDPE (72.31 kJ/m^2^). The highest impact strength among filled composites was observed in mineral wool recycled (40%) (11.04 kJ/m^2^), attributed to favourable filler–matrix interactions. Interestingly, soapstone-filled SS60 (5.27 kJ/m^2^) outperformed SS40, likely due to improved filler compaction at higher loadings. Conversely, gypsum-filled composites exhibited declining toughness with increased filler, confirming that higher mineral content compromises ductility.

The impact behaviour of particulate-filled RTCs is primarily governed by the stiffness–ductility balance between the filler and matrix. Inorganic fillers such as sand, fly ash, alumina, and soapstone tend to increase stiffness but reduce toughness due to their rigid, non-deformable nature, which limits plastic deformation and promotes stress concentration at the filler–matrix interface. When dispersion and adhesion are poor, these rigid inclusions act as crack initiators, resulting in reduced energy absorption and brittle fracture. Conversely, matrices with higher ductility, such as LDPE or PP, exhibit better impact retention because of their ability to deform plastically and dissipTABLEate energy around the filler particles. Optimised particle packing and improved interfacial bonding—as achieved with fine sand or treated fillers—enhance matrix confinement and resistance to crack propagation. The findings collectively indicate that maintaining uniform filler dispersion, moderate filler loading, and improved adhesion via compatibilisers is essential to mitigate the embrittling effect of rigid fillers and preserve the impact toughness of RTCs.

**Table 3:** Effects of particulate fillers on RTCs properties, their objectives, and outcomes across different matrix–filler systems are reported in the recent literature.

Type of Matrix	Type of Filler and Sizes	Content (wt.%)	Objectives of Research	Outcomes	Ref.
rHDPE	Mineral Wool (0.85–4 mm), Gypsum, Soapstone	0–60	Evaluate the mechanical and physical properties of rHDPE composites with various recycled particulate fillers.	Improved rigidity and moisture resistance; Tensile strength decreased by 39.4–66.2% and modulus increased by 24.5–102.1% compared.	[83]
rPP	Periwinkle Shell Powder (150–425 µm)	0–25	Investigate the effect of filler loading and particle size on mechanical properties of rPP composites	Tensile strength and modulus increased by 72.8% and 19.0% at 15 wt.% (150 µm), flexural strength increased by 40.3% at 20 wt.% (425 µm), and Shore A hardness improved by 69.2% at 25 wt.% (300 µm); excessive filler led to agglomeration and reduced strength	[94]
rHPPE/rPP	Silica Sand (100–300 µm)	60–80	Investigate the deformation and strength characteristics of highly filled sand–polymer composites derived from reclaimed thermoplastics.	Optimal compressive strength (≥25 MPa) retained up to 75 wt.% filler; surface treatment with stearic acid improved strength by 10–15%; addition of 0.1 wt.% silica nanoparticles enhanced strength by another 15%.	[105]
rPP	Fly Ash (76–152 µm)	1:1	Utilise industrial waste FA (coated by 0, 1, 2, 3, 5 gm of FP) as filler in rPP composites to enhance sustainability	Demonstrated flexural strength and modulus decreased by 8.3% and 29.6%, respectively, and increased by 6.4% and 1.4%; Impact strength increased by 100% and 53%, but decreased by 14% and 36%.	[106]
rPP	Graphene (500 μm)	0–2	Predict thermal and mechanical properties of rPP nanocomposites reinforced by graphene-based fillers	Young’s modulus and thermal conductivity showed 5.7–35.4% and 5.84% enhancement, respectively, and a 44.8% decrease in Poisson’s ratio.	[107]
rHDPE/ rLDPE	Silica Sand (<0.30 mm ≤1.35 mm)	50–83.3	Investigate the effect of sand particle size and content on mechanical and thermal properties of plastic-bonded sand composite	Produce the highest compressive strength, ranging from 65% and 80%; improved ductility, toughness, and thermal conductivity; suitable for paving tiles and partition walls.	[86]
rHDPE	Pine Dust (<250 μm ≤1000 μm)	0–30	Investigate the effect of pine dust particle size and content on the mechanical properties and water absorption of rHDPE composites	Tensile strength increased by 16.9% and 31.2% at 15% (500–1000 µm) and 20 wt.% (250–500 µm) particles; increased by 4.305% water absorption with 30 wt.% (<250) filler.	[97]
rPP	Waste printed circuit boards (<1 mm)	0–30	Develop sustainable composites using rPP and WPCB	Tensile strength increased and decreased by 0.49% (5 wt.%) and 6.34% to 27.56% (10–30 wt.%), respectively; impact strength decreased by 37.5% and 62.3% with 15% and 30% WPCB; enhanced thermal stability; effective use of electronic waste	[96]
rPET	Wollastonite, Mica, Talc (30 µm, 44 µm, 2.7 µm)	10–20	To evaluate mechanical, thermal, and morphological properties of rPET with single and hybrid mineral fillers for automotive applications.	Wollastonite- and talc-filled composites at 20 wt.% showed the highest flexural strength improvements of 22.8% and 16.7%, respectively. The addition of Mica (20 wt.%) increased tensile, compression, and flexural modulus by 91.1%, 123.6%, and 129.0%, respectively, while talc addition increased thermal flexural stability by 36.8%.	[108]
bPET	Waste Eggshell (66.74, 711.27, and 746.66 μm)	10	Evaluate how eggshell particle size affects the mechanical properties of bioPET composites.	Addition of 66.74 μm, 711.27 μm, and 746.66 μm sizes. Eggshell showed tensile strength changes of +5.94%, −0.77%, and −23.23%, with corresponding flexural strength reductions of 31.3%, 13.25%, and 11.78%. Flexural modulus increased by 29.45% (66.74 μm) and 10.46% (711.27 μm), but dropped by 6.77% (746.66 μm).	[93]

### 4.3. Durability Properties of Particulate-Filled RTCs

#### 4.3.1. Effect of Water Absorption

**Figure 13:**
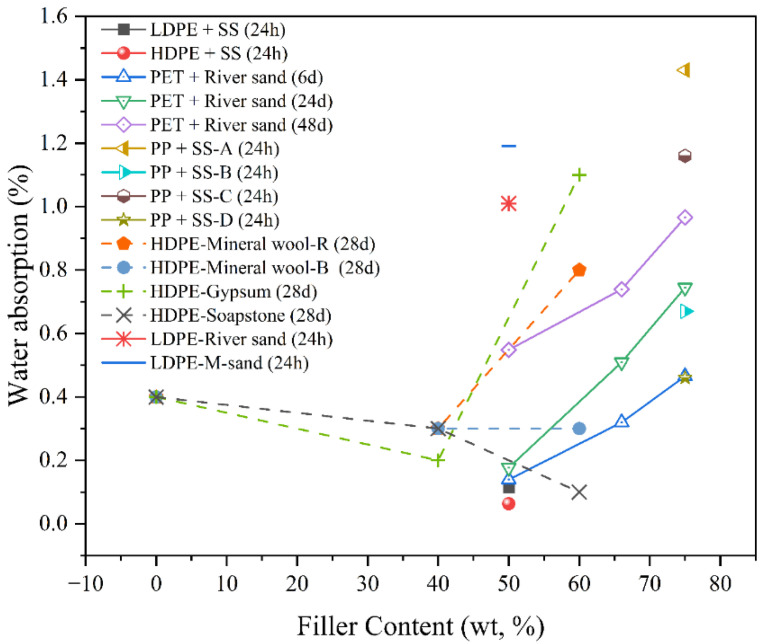
Effect of filler content on the water absorption of various RTCs, highlighting how increasing filler loading and type influence the hydrophobicity or permeability of the composite matrices [64,77,83,101,103].

Water absorption is a critical durability parameter for particulate-filled RTCs, as it directly affects dimensional stability, mechanical integrity, and interfacial bonding in moist environments, as presented in Figure 13. Fillers such as biochar, river sand, fly ash, CC and talc have been studied for their role in reducing water uptake by acting as diffusion barriers or altering the hydrophilicity of the matrix [109,110]. Soni et al. [103] explored the potential of waste plastics and silica sand. They reported that water absorption in sand–plastic composites was significantly influenced by the type of thermoplastic and filler content, with values ranging from 0.0397% to 0.1149%. The lowest water absorption (0.0397%) occurred in the composite containing 50 wt.% LDPE, 20 wt.% PET, and 30 wt.% sand, attributed to reduced porosity and the planar, impermeable nature of PET. Composites with HDPE and sand (50:50) showed moderate absorption (0.0634%), while the highest absorption was observed for the LDPE–sand (50:50) sample (0.1149%), due to LDPE’s lower density and less compact microstructure. The findings indicate that water absorption decreases with denser matrix formation, effective filler encapsulation, and thermoplastic properties that resist moisture ingress.

Similarly, Sormunen and Kärki [83] observed that water absorption in rHDPE composites was significantly influenced by the type and content of particulate fillers. Composites filled with inorganic particles such as gypsum and soapstone exhibited notably low water absorption values (≤1.1 wt.%), indicating improved moisture resistance. This behaviour was attributed to the dense packing and lower porosity of these fillers within the HDPE matrix, which limited pathways for moisture ingress. In contrast, composites containing wood particles showed higher water uptake due to the hydrophilic nature of lignocellulosic fillers. Babatunde et al. [77] investigated the water absorption behaviour of PET and sand composites at mix ratios of 1:1, 1:2, and 1:3. The results showed that water absorption remained below 1% across all mix proportions. This low absorption was attributed to the hydrophobic nature of PET, which coated sand particles and inhibited water ingress. However, water absorption increased slightly with higher sand content due to the hydrophilic property of sand and reduced PET availability for coating, as well as the presence of microcracks and increased porosity in higher sand mixes.

Mekideche et al. [64] reported that the water absorption of plastic-bonded sand composites using rPP varied with sand type, ranging from 0.46% for Khoubana sand (Mat D) to 1.43% for Oued Meitar sand (Mat A). The reduction in water absorption for Mat D and Mat B was attributed to their finer grain size and uniform distribution, which minimised porosity and interconnectivity between pores. The filler–thermoplastic mechanism influencing water absorption was governed by the degree of physical packing and encapsulation of sand grains by the PP binder, effectively limiting moisture ingress.

Mohan et al. [101] assessed the water absorption behaviour of rLDPE composites filled with river sand and manufactured sand. The river sand composite exhibited lower average water absorption (1.01%) compared to the manufactured sand composite (1.19%), indicating superior moisture resistance. The difference was attributed to better packing and reduced porosity in river sand mixtures. The filler–thermoplastic mechanism influencing water absorption was based on the LDPE’s inherent hydrophobicity and its encapsulation of filler particles, which limited water ingress through the composite structure.

The water absorption behaviour of RTCs is strongly influenced by the hydrophilicity and morphology of the filler. Inorganic fillers such as glass, fly ash, and sand exhibit minimal water uptake due to their non-polar, hydrophobic, and dimensionally stable surfaces, which act as diffusion barriers and reduce capillary transport through the matrix. In contrast, bio-fillers such as wood flour, rice husk ash, or agricultural residues contain hydroxyl groups that readily form hydrogen bonds with water, promoting interfacial debonding, swelling, and microvoid formation. Consequently, composites with inorganic fillers show improved dimensional stability and reduced permeability, whereas bio-filled systems require surface modification or coupling agents to limit water absorption and maintain structural integrity under humid or submerged conditions.

#### 4.3.2. Thermal Durability of Particulate-Filled RTCs

Thermal durability is a crucial factor influencing the long-term performance of particulate-filled RTCs in high-temperature environments. There are several studies that evaluated the effects of various particulate fillers on the thermal stability, flame retardancy, and heat resistance of RTCs. Salih et al. [111] assessed the flame-retardant properties of nano-CaCO_3_-filled LDPE/PP hybrid composites. The limited oxygen index value is higher for 9 wt.% (PPLL9) filler-loaded composites, which is 24.2% higher compared to composites with less than 9% filler. The burning rate measured is 10.23 mm/s for the 9 wt.% CaCO_3_-loaded composites, which is lower than compared of other samples.

**Figure 14:**
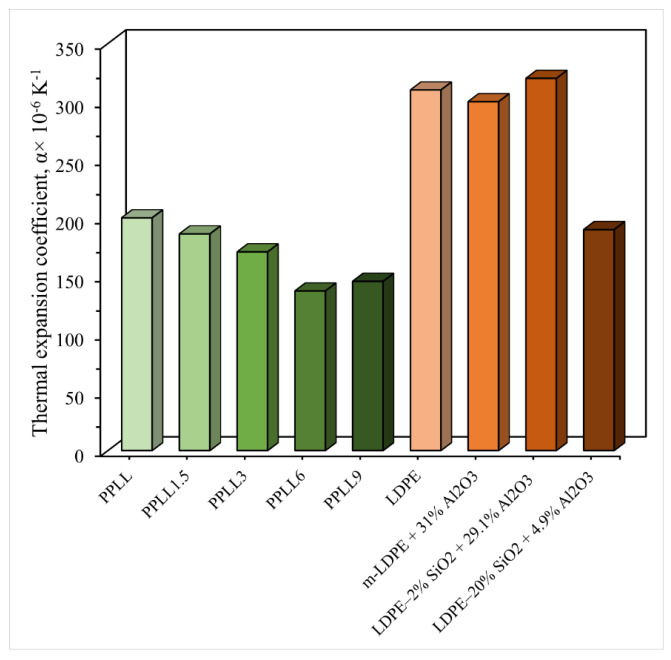
Effect of different fillers on the thermal expansion behaviour of recycled thermoplastic composites (RTCs), highlighting the reduction in thermal expansion coefficient with rigid inorganic fillers compared to unfilled matrices [111,112].

Additionally, the coefficient of thermal expansion decreased by ~31.4% compared to the PPLL sample, and by ~8.5% and ~37.1% for PP and LLDPE, respectively, as shown in Figure 14. The silica filler primarily affects the physical properties of the composites, including stiffness, modulus, wear and thermal resistance, hardness, and stability. Similarly, Olmos et al. [112] reported that neat LDPE exhibited an average coefficient of thermal expansion (CTE) of 310 × 10^−6^ K^−1^ with 0 wt.% alumina, consistent with literature values ranging from 1.8 × 10^−4^ to 4.0 × 10^−4^ K^−1^ [46]. Milling without nanoparticle addition (m-LDPE) resulted in a CTE of 300± 0.1 × 10^−6^ K^−1^ despite the incorporation of 31 wt.% alumina (Al_2_O_3_) from milling tool wear, indicating that the relatively low volume fraction of this low-CTE ceramic phase had little impact. Introducing silica nanoparticles markedly reduced the CTE: 2 wt.% SiO_2_ composites containing 29.1 wt.% alumina maintained a CTE of 320 ± 0.1 × 10^−6^ K^−1^, whereas 20 wt.% SiO_2_ composites with only 4.9 wt.% alumina achieved a ~40% reduction to 190 ± 0.1 × 10^−6^ K^−1^. The coefficient of thermal expansion (CTE) is presented in Figure 14. This substantial reduction closely matched predictions from the modified Levin model, attributed to the high surface-to-volume ratio and uniform dispersion of nanoparticles, which restrict polymer chain mobility in thin interparticle regions. The findings highlight the effectiveness of nanoscale ceramic fillers in tailoring LDPE’s thermal expansion to improve dimensional stability in thermo-mechanically demanding applications.

A drawback of thermoplastics is that they significantly soften upon heating. As this happens, their modulus decreases, leading to creep (gradual deformation over time), and at higher temperatures, they progressively lose their shape and then melt. There has been a great deal of effort spent in trying to overcome this limitation. Filling PP with talc is an early example of this. The addition of mineral fillers increases the modulus of all thermoplastics and increases their heat distortion temperature (HDT) [67]. Sadik et al. [19] investigated the thermal stability of rHDPE reinforced with WGP and observed that increasing WGP content significantly enhanced thermal resistance. The degradation temperature at 10% mass loss (T_10_%) increased from 444 °C for neat rHDPE to 467 °C for the composite containing 30 wt.% WGP, whereas the residual mass at 800 °C increased from 0.3% to 29.8%. This improvement was attributed to the low specific heat and thermal conductivity of WGP, which acts as a thermal insulator and physical barrier, hence slowing down polymer degradation. The findings confirm that the addition of WGP enhances the composite’s thermal insulation capacity and stability under elevated temperatures.

Furthermore, Babatunde et al. [77] examined the effect of elevated temperature (200 °C for 2 h) on the residual compressive strength of PET and sand composites. The post-heating strength reductions were 1.8 N/mm^2^, 1.5 N/mm^2^, and 1.6 N/mm^2^ for mix ratios 1:1, 1:2, and 1:3, respectively. The higher loss in the 1:1 mix was attributed to the greater PET content, given that PET possesses low fire resistance. The sand acted as a thermal insulator, reducing degradation. The 1:2 mix exhibited minimal reduction in strength, indicating an excellent interaction between the binder and filler under thermal exposure. Moulai Arbi et al. [100] reported that the thermal stability of PET improved significantly with the addition of brick sand, with the degradation temperature increasing from 220 °C to 314 °C at a 45% brick sand content. This enhancement was attributed to the formation of a protective barrier by the dispersed brick particles, which delayed the diffusion of volatile degradation products. The filler–thermoplastic mechanism influencing thermal behaviour involved the insulating effect and dispersion quality of the brick particles within the PET matrix.

On the other hand, Mohan et al. [101] reported that the thermal properties of rLDPE composites were influenced by the type of sand filler used. The thermal conductivity was lower for river sand composites (0.67 W/m·K) compared to manufactured sand composites (0.72 W/m·K) due to better packing and reduced internal voids in the river sand mixture, which improved thermal insulation. Similarly, the volumetric thermal expansion coefficient was slightly higher for river sand composites (4.23 mK^−1^) than for manufactured sand composites (4.13 mK^−1^), with the latter showing reduced expansion due to denser packing and thermal restraint. The filler–thermoplastic mechanisms influencing both properties were driven by the distribution, interfacial contact, and packing of sand particles within the LDPE matrix, which controlled heat transfer pathways and constrained thermal deformation. Bou-Kandil et al. [78] indicated that the thermal stability of HDPE nanocomposites improved slightly with the incorporation of ZnO nanoparticles, resulting in an increase in onset degradation temperature by approximately 15–20 °C compared to pure HDPE. This enhancement was attributed to the stabilising effect of ZnO, which hindered thermal decomposition. The filler–thermoplastic mechanism influencing thermal stability was governed by the nanoparticle-induced barrier effect and improved interfacial interaction, which slowed down the thermal degradation process under a nitrogen atmosphere. Zhao et al. [113] investigated the thermal stability and flammability of HDPE composites filled with rice husk (RH) and found that RH addition delayed HDPE’s thermal degradation by approximately 40 °C and significantly reduced peak heat release rate (PHRR) by up to 58% at 50 wt.% RH content. The improvement in thermal and fire resistance was attributed to the formation of a silica-based protective layer from RH decomposition, which acted as a heat shield and diffusion barrier during combustion.

The filler–thermoplastic mechanism was governed by the generation and integrity of this silica layer, which restricted oxygen access and reduced degradation, resulting in enhanced thermal and flame-retardant properties.

The thermal durability of RTCs depends on the filler’s thermal conductivity, specific heat, and degradation resistance. Inorganic fillers such as fly ash, talc, and alumina enhance heat resistance by acting as heat sinks and improving load-bearing capacity at elevated temperatures. Their high melting points and stable crystalline structures suppress polymer softening and delay the onset of thermal degradation. Conversely, bio-fillers decompose at lower temperatures, releasing volatiles that accelerate polymer oxidation and chain scission. The improvement in thermal stability of inorganic-filled composites reflects their ability to restrict molecular motion and stabilise the polymer matrix during heating, confirming their suitability for applications exposed to cyclic or sustained thermal loading.

#### 4.3.3. Effect of Solar Ultraviolet (UV) Radiation

**Figure 15:**
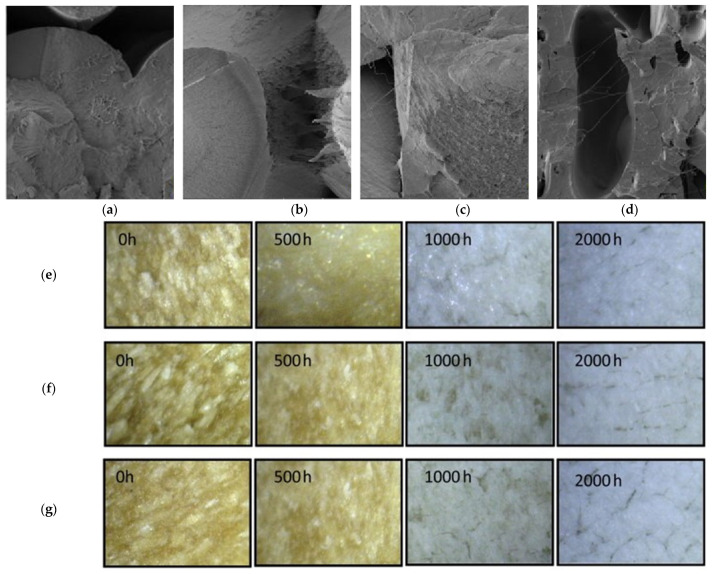
UV degradation behaviour of recycled thermoplastic composites (RTCs) and biocomposites: (**a**–**d**) morphological evolution of PLA and PLA_CG after 0–12 weeks of UV exposure, showing surface cracking and colour change; (**e**–**g**) comparison of unmodified and chemically modified wood–plastic composites (WPCs) demonstrating improved UV resistance with acetylation and propionylation treatments [115,116].

**Figure 16:**
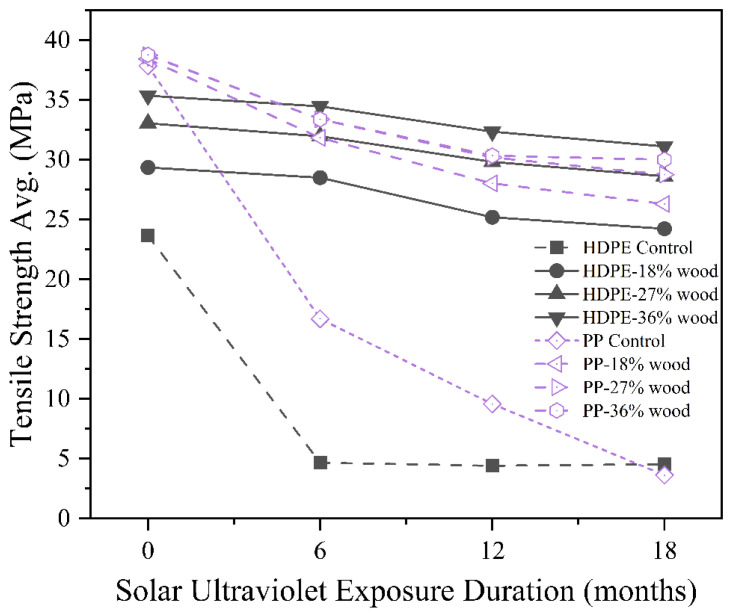
Effect of fillers on the average tensile strength of recycled thermoplastic composites (RTCs) after solar ultraviolet (UV) exposure, showing the retention or loss of tensile properties based on filler type and interfacial stability under photodegradation [117].

Prolonged solar ultraviolet (UV) exposure leads to polymer chain scission, discolouration, surface cracking, and loss of strength [114], making UV resistance an essential aspect of RTC durability in outdoor applications. A morphological analysis revealed that UV exposure induced severe surface degradation in neat PLA, with smooth fracture surfaces becoming rough, porous, and microcracked after 12 weeks. In contrast, PLA filled with 10 wt.% coffee grounds (CG) exhibited fewer cracks and retained more cohesive morphology, suggesting that the filler acted as a UV barrier and delayed photo-oxidative chain scission (Figure 15a–d) [115]. Figure 15e–g shows microscopic images of WPC samples after different durations of prolonged UV exposure, revealing progressive surface whitening with exposure time. The control poplar WPC exhibited significant fibre loss and whitening after 1000–2000 h, whereas acetylated and propionylated fibre-based WPCs demonstrated improved photostability with less surface degradation [116]. Siddiqui et al. [117] examined the effect of extensive solar ultraviolet irradiation on the tensile strength of wood–plastic composites (WPCs) based on HDPE and PP, as presented in Figure 16. Injection-moulded samples with wood fibre loadings from 0 to 36 wt.% were exposed to both over 18 months of natural weathering and up to 1180 h of accelerated laboratory UV exposure. The results showed that tensile strength decreased approximately linearly with exposure duration, with unfilled PP and HDPE exhibiting more rapid degradation than wood-filled composites.

Notably, HDPE-WPCs retained tensile strength better than PP-WPCs. The improved UV stability was attributed to the light-shielding effect of wood fibres and the stabilising role of phenolic lignin, which delayed photo-oxidative degradation. Optimal UV resistance was observed at 18 wt.% wood loading, above which no significant additional benefit was noted. Moreover, UV exposure significantly influenced the modulus of elasticity, which increased progressively with UV exposure duration due to photochemical crosslinking and increased crystallinity in the polymer matrix. In contrast, the elongation at break declined sharply across all formulations, primarily due to interfacial debonding and adhesive failure at the wood–polymer interface.

Similarly, surface hardness measured by the Shore D scale decreased with exposure time, particularly in control samples without wood filler. Wood fibres acted as UV shields and contributed phenolic compounds that stabilised the matrix, especially in HDPE composites, slowing mechanical deterioration. These effects were most pronounced in formulations containing 18 wt.% wood, which provided an optimal balance between UV resistance and mechanical integrity [117]. Abou-Kandil et al. [78] reported that the UV shielding efficiency of HDPE nanocomposites was significantly enhanced by the incorporation of ZnO nanoparticles, with maximum absorption observed for particles calcinated at 350 °C (sample Z4, ~25 nm). This formulation (2.5 wt.% ZnO) exhibited strong UV absorption while maintaining high visible light transparency, making it ideal for UV-protective packaging applications. The filler–thermoplastic mechanism influencing UV performance was attributed to the dual effect of UV absorption and light scattering by uniformly dispersed ZnO nanoparticles within the HDPE matrix. Wang et al. [118] indicated that the tensile strength of HDPE/titanium dioxide (TiO_2_) composites decreased slightly after accelerated ultraviolet (UV) irradiation. Among the composites, the unirradiated HDPE exhibited the highest tensile strength (31.97 MPa), while the HDPE/mixed crystal TiO_2_ composite (PETM) showed the lowest (24.67 MPa) due to reduced compatibility. The degradation in tensile strength after UV exposure was attributed to polymer chain scission and surface cracking, especially in the amorphous regions. The filler–thermoplastic mechanism influencing tensile strength was governed by TiO_2_ particle distribution and UV-induced structural changes, wherein chain breakage and oxidative degradation reduced the material’s mechanical integrity.

The UV resistance of RTCs is largely governed by the filler’s optical and chemical characteristics. Inorganic fillers such as glass, fly ash, and mineral oxides act as UV shields by scattering and reflecting ultraviolet radiation, thereby reducing surface oxidation, colour fading, and chain scission in the polymer matrix. These fillers also absorb part of the UV energy, converting it to harmless thermal energy and preventing photodegradation. In contrast, bio-fillers accelerate UV ageing due to the presence of lignin and cellulose, which absorb UV light and promote radical formation and oxidative reactions at the interface. Thus, composites with inorganic fillers demonstrate superior colour retention, mechanical stability, and surface integrity under prolonged UV exposure.

## 5. RTCs in Civil Engineering: Current Applications, Challenges, and Limitations

Despite increasing research interest and significant progress in the development of RTCs, their mechanical properties often fail to meet the stringent requirements of structural civil engineering applications. For instance, RTCs reinforced with commonly used fillers such as sand, wood flour, or agricultural residues typically exhibit tensile and flexural strengths that are considerably lower than those of conventional construction materials. Ferdous et al. [119] demonstrated that certain commercial polymer composite sleepers achieved tensile strengths in the range of 17.2–20.6 MPa and moduli of elasticity around 1.5–1.8 GPa, significantly lower than softwood timber sleepers, which can reach tensile strengths of approximately 49.3 MPa and a modulus of 7.4 GPa. These disparities underscore the mechanical shortcomings of RTCs in high-load applications, such as railway sleepers and structural building components.

Adding to these challenges, the inherent variability of recycled thermoplastics, stemming from inconsistent feedstock quality, contamination, and thermal degradation during recycling, results in unpredictable mechanical behaviour and reduced reliability [22]. Although high-performance composites reinforced with continuous fibres or hybrid filler systems have been developed to address these limitations, their adoption is often constrained by higher production costs, complex manufacturing processes, and limited scalability for large-scale applications [120]. Table 4 summarises their key findings, challenges and limitations of representative studies on particulate-filled RTCs in structural applications, detailing filler type, particle size (macro, micro, or nano), and corresponding effects on mechanical performance. Furthermore, the lack of standardised design codes and mechanical benchmarks for RTCs has impeded their widespread acceptance and integration into structural applications.

Recent innovations, such as compatibiliser-treated hybrids and nanofiller-modified systems show promise in narrowing the performance gap [125]. However, trade-offs persist between cost, performance, and sustainability. Consequently, there is an urgent need for further research into cost-effective processing techniques, enhanced interfacial adhesion between matrix and fillers, and thorough long-term durability assessments under realistic civil engineering loading conditions. Addressing these gaps will be critical to unlocking the full potential of RTCs as sustainable alternatives in structural applications.

## 6. Emerging Applications and Future Recommendations

In response to these challenges, polymer composites have emerged as a cornerstone of modern materials science, providing sustainable solutions to reduce landfill dependency. Different polymer composites are utilised across various sectors, including agriculture, the built environment, electrical and electronics, automotive, aerospace, construction, packaging, and household goods. Among these, thermoplastic composites represent a particularly versatile class of materials, with lower manufacturing costs, recyclability, processability, and the ability to enhance properties through strategic reinforcement [126]. The global thermoplastic composites market was valued at around USD 22.2 billion in 2021 and is predicted to grow to USD 31.8 billion by 2027, exhibiting a CAGR of 6.2% throughout the forecast period [127].

Recycled thermoplastic composites, particularly those reinforced with cost-effective fillers such as waste glass sand, offer diverse potential across civil engineering applications. Given their favourable properties, such as corrosion resistance, low maintenance, and high durability in aggressive environments, several promising application areas can be highlighted:

- Structural elements under dynamic loads: RTCs can be engineered as sustainable alternatives to hardwood and prestressed concrete railway sleepers, where repeated impact and cyclic stresses govern long-term performance.
- Road and bridge construction: Non-structural elements such as formwork, guardrails, deck panels, and retaining walls, where RTCs can replace wood, steel, or concrete.
- Coastal and Hydraulic Structures: Suitable for piers, embankment facings, and flood barriers due to hydrophobicity and biological resistance.
- Urban Furniture and Landscape Design: Used in outdoor furniture, fencing, decking, and boardwalks, especially in parks and recreational zones, because they offer longevity and recyclability.
- Underground Utility Infrastructure: Composite pipes, cable ducts, and drainage systems made from filled RTCs offer resistance to chemical leaching and long-term durability in soil environments.

The following recommendations are proposed to enhance the structural reliability and environmental value of particulate-filled RTCs in civil and structural engineering:

- Novel waste-derived fillers and hybrids: Beyond conventional waste glass and mineral sand, underexplored fillers such as silica sand, fly ash, ceramic waste, and industrial by-products should be incorporated to enhance stiffness, dimensional stability, and environmental value.
- Optimised filler–matrix interface: Research should focus on compatibilisers, surface modification, and particle tailoring (size, aspect ratio, dispersion) to enhance stress transfer and ensure durability without compromising processability.
- Few studies report fatigue, creep, UV degradation, hygrothermal ageing, or freeze–thaw resistance of RTCs. Moreover, the combined influence of environmental factors—such as UV exposure, moisture absorption, and thermal cycling—remains largely unexplored. Systematic laboratory protocols, incorporating both accelerated and coupled environmental exposure, together with field validation, are needed to establish reliable design safety margins.
- Circular economy integration: Future work should extend beyond mechanical performance to include recyclability after service, embodied CO_2_, and comprehensive LCA benchmarks for RTCs in real infrastructure.
- Cost-performance optimisation: Innovative processing routes and filler optimisation must be evaluated not only for strength gains but also for economic feasibility relative to timber, steel, and concrete alternatives.
- Standardisation and design guidelines: Development of international standards, testing protocols, and design codes is crucial for the large-scale adoption of RTCs in industry and infrastructure projects.
- Data-driven and modelling-based design: Future studies should integrate AI-assisted optimisation, multiscale modelling, and predictive durability simulations to correlate filler characteristics with composite performance. Machine learning frameworks can accelerate the design of hybrid and surface-activated fillers, enabling tailored interfacial bonding, improved long-term durability, and reduced experimental dependency through model-guided material development.

## 7. Conclusions

This review has synthesised current knowledge on the manufacturing, characterisation, and performance optimisation of particulate-filled recycled thermoplastic composites (RTCs) by highlighting the mechanistic role of fillers in compensating for the degradation that occurs during polymer recycling. Repeated recycling of thermoplastics leads to chain scission, oxidation, and loss of stabilisers, resulting in reductions of 30–70% in ductility, reductions of 15–40% in strength, and decreased thermal stability relative to virgin polymers. Particulate fillers provide a viable strategy to compensate for these degradation effects by improving stiffness, dimensional stability, and durability when properly engineered.

Inorganic fillers such as waste glass, minerals, ceramics, and silica generally increase the tensile modulus by 10–200% and flexural modulus by 20–150%, and enhance thermal resistance and dimensional stability. However, due to their rigidity and poor deformability, they often reduce elongation at break by 30–90% unless compatibilisers or surface treatments are used. Organic fillers (e.g., wood flour, rice husk ash) improve toughness and reduce composite density but may lead to strength losses if interfacial bonding is insufficient. Nano-engineered fillers (ZnO, nano-silica, graphene) provide dual reinforcement—improving modulus, barrier resistance, and UV stability even at low loadings—though they require excellent dispersion to avoid agglomeration.

Durability improvements were also evident across studies, with fillers reducing water absorption by 20–50%, lowering thermal expansion coefficients by 15–40%, and improving UV stability due to crack-blocking and shielding mechanisms. These enhancements are strongly governed by filler morphology, surface chemistry, and dispersion state, which together determine stress transfer efficiency, crack initiation behaviour, and long-term stability.

The review emphasises that the effectiveness of fillers is governed by design variables including particle size, shape, aspect ratio, surface chemistry, and dispersion—each controlling fracture behaviour, load transfer efficiency, and resistance to environmental degradation. These mechanistic insights point to the need for tailored interface engineering and optimisation of filler loading to avoid brittle failure while maintaining manufacturability.

Emerging research indicates a growing need for exploring next-generation sustainable, waste-derived, and nano-engineered fillers, improving interfacial tailoring, and developing predictive models for long-term durability under environmental loads. Advancing these areas will enable RTCs to be more reliably designed for non-structural and semi-structural civil engineering applications, while establishing the groundwork needed for eventual structural use.

## Figures and Tables

**Table 1:** Summary of key physical, thermal and mechanical properties of different thermoplastics relevant to the design of particulate-filled RTCs [38,39,40,41,42,43,44,45,46,47,48,49,50,51,52,53].

Polymers	ρ (g/cm^3^)	E (GPa)	T_g_ (°C)	T_m_ (°C)	T_p_ (°C)
PP	0.899–0.920	0.95–1.776	−23 to −10	160–176	200–290
LDPE	0.910–0.925	0.1–0.38	–125	105–116	150–230
HDPE	0.941–1.000	0.41–1.49	−133 to −100	120–140	150–290
PA-6	1.09–1.14	2.9	40–48	215–216	215–270
PA-66	1.08–1.14	2.5–3.9	50–80	250–269	250–320
PC	1.19–1.24	2.3–3.0	140.5–150	–	260–330
PBT	1.23–1.35	2.37	20–45	224–240	246–290
PET	1.30–1.40	2.7–4.0	69–110	246–265	256–310
PEEK	1.264–1.32	3.1–3.8	139–153	334–343	370–400
PPS	1.30–1.40	2.6–3.9	85–95	275–290	300–340
PEI	1.27–1.28	3	215–225	–	330–420
PAI	1.38–1.45	2.8–4.4	244–290	–	340–400

Notes: density (ρ); tensile modulus (E); glass transition temperature (T_g_); melt point (T_m_); process temperature (T_p_).

**Table 4:** Representative studies on particulate-filled RTCs for structural and civil engineering applications, including filler type, particle size, and corresponding mechanical and durability behaviour.

Matrix Types	Filler Types and Sizes	Applications	Key Findings	Challenges and Limitations	Refs.
rHDPE	Waste glass (<50 μm and <200 μm)	Marine structures such as docks and sea barriers	At this optimum ratio, flexural, tensile, and compressive strengths of 33.3 MPa, 19.6 MPa, and 12.8 MPa, respectively; compatibilisers improved filler dispersion.	Processing limits with high filler content; Durability: UV radiation, temperature fluctuations, and long-term loading remain unproven; limited field validation.	[121]
Recycled PET, PE-HD, PE-LD, PP and PS	Steel-strengthened and un-strengthened	Wall panels, non-load-bearing materials, and Eco-building materials	Steel reinforcement enhanced strength and stiffness by over 300% without loss of ductility, achieving suitable panel performance, though still lower than traditional materials like timber.	Low bearing capacity; not suitable for major load-bearing applications.	[122]
rPE	Recycled tyre (0.25–0.5 mm)	Road and paving materials	Si69 treatment improved tensile stress by 34% and strain by 70%, while MAPE enhanced strain by 47% with negligible strength change. VTMS treatment slightly reduced both stress and modulus compared to untreated samples.	Difficulties in consistent blending; thermal degradation risk.	[123]
rPE	Sand (>2 mm)	Structural blocks, precast, and Modular construction materials.	Flexural strength increased from 8 MPa at 4% filler to 30 MPa at 25% filler, representing a 275% improvement, exceeding that of many steel-reinforced concretes.	Brittle failure at low binder ratios; thermal expansion mismatch.	[102]
rHDPE	Mineral fillers (wool, gypsum, soapstone)	Structural construction boards	Enhanced stiffness and moisture resistance; usable for semi-structural applications.	Poor tensile strength; filler incompatibility; not suitable for heavy structural loads; variation in filler properties impacts consistency.	[83]
Municipal waste plastic	Coal ash (0.1–1000 μm)	Sustainable Railway sleepers	Tensile stress and modulus improved from 6.8 MPa to 19.0 MPa, and from 755 MPa to 2281 MPa, a 179% and 202% increase; Compressive and flexural strength improved by 74% and 66%, respectively. Up to 60% coal ash filler yielded suitable mechanical strength for sleeper use.	Long-term durability and creep under load remain concerns; it requires more field validation.	[124]
Recycled plastic composites (Type-1)	Particle filler	Railway sleepers	Recycled plastic sleepers offer environmental benefits and ease of handling, but new Australian hardwood sleepers show a MoR of 47–110 MPa, while most composite sleepers exhibit much lower strength and stiffness.	Low anchorage capability, void formation, creep deformation, UV and moisture degradation, and absence of long-term performance standards.	[28]
		Alternative railway sleepers	Recycled plastic sleepers with a bending modulus below 1.0 GPa showed W-shaped deflection and ~42% higher rail seat deformation compared with timber sleepers (MoE = 13.0 GPa).	Low MoE sleepers cause stress concentrations and unstable deflection; recycled plastic sleepers deform excessively.	[23]

## Data Availability

No new data were created or analyzed in this study.
